# Multi-Target Angle Tracking Algorithm for Bistatic MIMO Radar Based on the Elements of the Covariance Matrix

**DOI:** 10.3390/s18030805

**Published:** 2018-03-07

**Authors:** Zhengyan Zhang, Jianyun Zhang, Qingsong Zhou, Xiaobo Li

**Affiliations:** National University of Defense Technology, Hefei 230037, China; zjy921@sina.com (J.Z.); zqseei@163.com (Q.Z.); lxb_eei@163.com (X.L.)

**Keywords:** bistatic multiple input multiple output radar, covariance matrix, angles tracking, least square method, high precision

## Abstract

In this paper, we consider the problem of tracking the direction of arrivals (DOA) and the direction of departure (DOD) of multiple targets for bistatic multiple-input multiple-output (MIMO) radar. A high-precision tracking algorithm for target angle is proposed. First, the linear relationship between the covariance matrix difference and the angle difference of the adjacent moment was obtained through three approximate relations. Then, the proposed algorithm obtained the relationship between the elements in the covariance matrix difference. On this basis, the performance of the algorithm was improved by averaging the covariance matrix element. Finally, the least square method was used to estimate the DOD and DOA. The algorithm realized the automatic correlation of the angle and provided better performance when compared with the adaptive asymmetric joint diagonalization (AAJD) algorithm. The simulation results demonstrated the effectiveness of the proposed algorithm. The algorithm provides the technical support for the practical application of MIMO radar.

## 1. Introduction

In recent years, the multiple-input multiple-output (MIMO) radar has been proposed as a new system radar [1]. Multiple array elements of the MIMO radar can transmit mutually orthogonal waveforms, which have a high degree of freedom [2,3,4]. Compared with the phased array radar, the MIMO radar has more accurate performance in target detection, identification, parameter estimation, and tracking. According to the array element configuration, the MIMO radar is divided into the statistical MIMO radar and coherent MIMO radar [5]. The array elements of the statistical MIMO radar are far away from each other, therefore, the statistical MIMO radar obtains spatial diversity gain, which can effectively improve the estimate performance of the scintillation target. The transmit and receive elements of the coherent MIMO radar are closely spaced, which can effectively improve the estimation accuracy of the target parameters, increase the number of the maximum identification targets, and so on. In the coherent MIMO radar, the bistatic MIMO radar is an important structure. The bistatic MIMO radar, which combines the advantages of MIMO radar and bistatic radar, effectively reduces the requirement of the three synchronizations (time, space, frequency). Therefore, the bistatic MIMO radar was used as the research object in this paper.

The existing parameter estimation algorithms in the bistatic MIMO radar are mostly aimed at stationary targets [6,7,8,9,10,11,12,13,14,15,16], which contain the estimation of signal parameters via rotational invariance technique (ESPRIT) algorithms [6,7,8,9], the Capon algorithm [10], the multiple signal classification (MUSIC) algorithms [11,12,13,14,15,16], and so on.

However, when the target is moving, the performance of the algorithm in [6,7,8,9,10,11,12,13,14,15,16] will decrease or even fail, which cannot be applied for tracking moving targets. The algorithms described above are all based on feature subspaces and require eigendecomposition. The computational complexity of the eigendecomposition process for the N dimension square matrix is generally $o\left(N^{3}\right)$. The covariance matrix obtained in bistatic MIMO radar has a large dimension, which is the product of the number of transmitters and receivers. Therefore, the computational complexity required for eigendecomposition is large and the project realization is more difficult. In addition, these eigendecomposition and eigensubspace methods are a class of batch processing methods. Obviously, these algorithms cannot be applied to time-varying signals, but in the actual battlefield environment, the goal often moves. The target angle tracking is the key problem that restricts the practical application of bistatic MIMO radar. Therefore, this paper studies the angle tracking problem in bistatic MIMO radar.

There are some published studies on MIMO radar tracking. The monostatic MIMO radar tracking algorithm is given in [17], which has low complexity, but the cost is the reduction of tracking performance. In [18], Kalman was introduced into the projective approximation subspace tracking with deflation (PASTd) algorithm. The Kalman filter was used to realize data association, and the algorithm converges quickly. In [19], a low-complexity angle tracking algorithm in monostatic MIMO radar was proposed. The studies are all about target tracking for the monostatic MIMO radar.

Bistatic MIMO radar is different from monostatic MIMO radar and consists of a transmitting and receiving base. The corresponding direction of arrivals (DOA) and direction of departure (DOD) are not equal. Therefore, the joint steering vector is more complicated. The above algorithms in [17,18,19] cannot solve the problem of target tracking in bistatic MIMO radar.

In [20], the PASTd algorithm in the array-signal-processing was introduced to the bistatic MIMO radar, and the target tracking problem of the MIMO radar was successfully solved. However, it requires an additional data correlation operation and cannot track the targets of the same DOD or DOA. In order to solve the deficiency of [20], reference [21] proposed a low complexity tracking algorithm in bistatic MIMO radar. The algorithm deduces the formula of the covariance matrix difference of an adjacent moment, and then achieves the target angle tracking. However, the algorithm from [21] uses only partial covariance matrix information, and the tracking performance is low. A target tracking algorithm based on Adaptive Asymmetric Joint Diagonalization (AAJD) was proposed in [22]. The algorithm does not need an additional correlation operation and can track the targets whose DOD or DOA is the same. Nevertheless, the performance of the algorithm is reduced by reusing the estimation angle of the last time.

In this paper, we consulted the monostatic MIMO radar angle tracking idea in [19] to propose a DOD and DOA tracking algorithm suitable for bistatic MIMO radar. The covariance matrix, constructed by the monostatic MIMO radar signal, satisfied the Toeplitz form in [19]. However, the DOD and DOA were different and the covariance matrix did not satisfy the Toeplitz structure in the bistatic MIMO radar. Therefore, we proposed an improved DOA and DOD tracking method based on three approximations. Then, we found that the covariance matrix satisfied the approximate Toeplitz property, whose partial elements in a straight line paralleled to the principal diagonal were equal. On this basis, our algorithm used the approximate Toeplitz property to take the average operation, which was equivalent to improve the signal-to-noise ratio (SNR). The proposed algorithm could realize automatic matching and association of DOD and DOA. Error analysis was also derived in this paper. Finally, the simulation results were presented to verify the effectiveness of the proposed algorithm.

There were some differences between the algorithm in [19] and the proposed algorithm. (1) Reference [19] proposed a low-complexity angle tracking algorithm in monostatic MIMO radar. However, we solved the DOD and DOA tracking problems of the bistatic MIMO radar. (2) The monostatic MIMO radar only needed to estimate DOA, but the algorithm in this paper needed to solve the DOD and DOA estimation, therefore the problem is more complicated. In this paper, the algorithm extended the approximate idea in [19] and solved the target angle by three approximation operations. (3) The monostatic MIMO radar angle tracking algorithm requires that the covariance matrix satisfy the Toeplitz form. Since the DOD and DOA are different, the covariance matrix is more complicated and does not satisfy the Toeplitz form in bistatic MIMO radar. Therefore, the algorithm in [19] cannot be used to solve the problem directly in this paper. So we used the approximate Toeplitz properties to improve the tracking performance.

Thus, the algorithm cannot only be seen as an extension of the work in [19] but is also an improved algorithm. The simulation results showed that the proposed algorithm had a better tracking performance than the angle tracking algorithm in [19,20,21,22].

The rest of this paper is organized as follows. In Section 2, the signal model of bistatic MIMO radar is presented. Section 3 establishes our angle tracking algorithm based on the elements of the covariance matrix of the receive signal. Section 4 compares the performance of the algorithm in [19,20,21,22] and our algorithm. The simulation results verify the effectiveness of the proposed algorithm. Finally, Section 5 concludes the paper.

*Notations*: ${\left(•\right)}^{T}$, ${\left(•\right)}^{H}$, ${\left(•\right)}^{+}$, and ${\left(•\right)}^{-1}$ denote the transpose, Hermitian transpose, pseudoinverse and inverse operations, respectively. $I_{K}$ is an K×K identity matrix; vec(•) is the vectorization of a matrix; diag(υ) stands for diagonal matrix whose diagonal is a vector v; ⊗ and ⊕ are the Kronecker product and Hadamard product, respectively.

## 2. Signal Model

**Figure 1:**
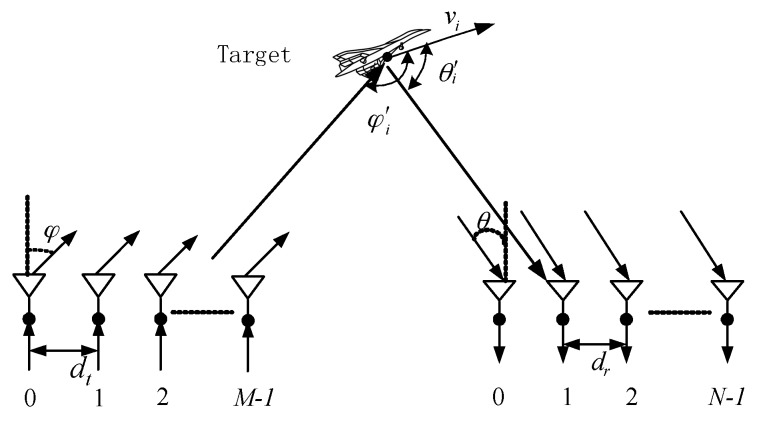
Bistatic multiple-input multiple-output (MIMO) radar transceiver element configuration.

In this paper, a bistatic MIMO radar was used to observe the moving targets in the air. The distances between the targets and bases are far, so the target satisfies the point target model. The bistatic MIMO radar is composed of M transmit antennas and N receive antennas. The space between the transceiver antennas is the same and half of the wavelength. The configuration of the bistatic MIMO radar is shown in Figure 1.

It is assumed that there is a P far-field moving point target in the air, and the DOD and DOA at time t is $\left[\left(\varphi_{t,1},\theta_{t,1}\right),\left(\varphi_{t,2},\theta_{t,2}\right),\cdots \left(\varphi_{t,P},\theta_{t,P}\right)\right]$, respectively. The velocity of the target i is $v_{i}$, and the angles between the moving direction and the DOD and DOA directions are $\varphi_{i}^{\prime }$ and $\theta_{i}^{\prime }$, respectively.

The transmit signal radiates to P targets, and the signal that arrives at the receive elements after scattering is

(1) $$\bar{x}\left(t\right)=\sum_{i=1}^{P}a_{r}\left(\theta_{t,i}\right)\varepsilon_{i}a_{t}^{T}\left(\varphi_{t,i}\right)s\left(t-\tau_{i}\right)\exp\left(j\omega_{i}t\right)+\bar{n}\left(t\right)$$

where $\omega_{i}=2\pi \frac{v_{i}\left(\cos\theta_{i}^{\prime }+\cos\varphi_{i}^{\prime }\right)}{\lambda }$; $f_{i}=\frac{v_{i}\left(\cos\theta_{i}^{\prime }+\cos\varphi_{i}^{\prime }\right)}{\lambda }$ is the doppler shift. The receive steering vector is $a_{r}\left(\theta_{t,i}\right)={\left[1,e^{j\pi \sin\theta_{t,i}},\cdots ,e^{j\pi \left(N-1\right)\sin\theta_{t,i}}\right]}^{T}$; and the transmit steering vector is $a_{t}\left(\varphi_{t,i}\right)={\left[1,e^{j\pi \sin\varphi_{t,i}}\cdots ,e^{j\pi \left(M-1\right)\sin\varphi_{t,i}}\right]}^{T}$. $\varepsilon_{1},\cdots ,\varepsilon_{P}$ is the scattering coefficient of the observation target and satisfies the Swerling II model, which is invariable within the pulse time. $s\left(t-\tau_{i}\right)=[s_{1}\left(t-\tau_{i}\right)\exp\left[j2\pi f_{c}\left(t-\tau_{i}\right)\right],\cdots ,s_{M}\left(t-\tau_{i}\right){\exp\left[j2\pi f_{c}\left(t-\tau_{i}\right)\right]]}^{T}$, $\bar{n}\left(t\right)$ is Gaussian additive white noise.

The signal $\bar{x}\left(t\right)$ is filtered through a set of matched filters, let each filter match only one transmit signal. Let the delay of matched filter of ith target signal is $\tau_{i}^{\prime }$, and $\tau_{i}^{\prime }=\tau_{i}$.

The output of the signal in Equation (2) after matched filtering is

(2) $$\begin{array}{cc} x\left(t\right) & =\sum_{i=1}^{P}a_{r}\left(\theta_{t,i}\right)\varepsilon_{i}a_{t}^{T}\left(\varphi_{t,i}\right)s\left(t-\tau_{i}\right)\exp\left(j\omega_{i}t\right)s^{*}\left(t-\tau_{i}^{\prime }\right)+\bar{n}\left(t\right)s^{*}\left(t-\tau_{i}^{\prime }\right)\\ & =\sum_{i=1}^{P}a_{r}\left(\theta_{t,i}\right)\varepsilon_{i}\exp\left(j\omega_{i}t\right)a_{t}^{T}\left(\varphi_{t,i}\right)+n\left(t\right) \end{array}$$

$\varepsilon_{i}\exp\left(j\omega_{i}t\right)$ and $\varepsilon_{i}$ has the same statistical properties, $\varepsilon ={\left[\varepsilon_{1}\exp\left(j\omega_{1}t\right),\cdots ,\varepsilon_{P}\exp\left(j\omega_{P}t\right)\right]}^{T}$ still satisfy the Swerling II model. The mean and variance of n(t) is 0 and $\sigma^{2}$.

Further simplify Equation (3),

(3) $$x\left(t\right)=A_{r}\left(\theta \right)diag\left(\varepsilon \right)A_{t}^{T}\left(\varphi \right)+n\left(t\right)$$

Consider the vectorization of x(t) in (4),

(4) $$y\left(t\right)=A_{t}\left(\varphi \right)⊙A_{r}\left(\theta \right)\mathrm{vec}\left(diag\left(\varepsilon \right)\right)+n\left(t\right)=W_{t}\left(\varphi ,\theta \right)\varepsilon +n\left(t\right)$$

where $W_{t}\left(\varphi ,\theta \right)=[a_{r}\left(\theta_{t,1}\right)⊗a_{t}\left(\varphi_{t,1}\right),a_{r}\left(\theta_{t,2}\right)⊗a_{t}\left(\varphi_{t,2}\right),\cdots ,a_{r}\left(\theta_{t,P}\right)⊗a_{t}\left(\varphi_{t,P}\right)]$ denotes MN×P joint steering vector.

Consider

(5) $$R_{t}=E\left[y\left(t\right)y^{H}\left(t\right)\right]=W_{t}R_{\varepsilon }W_{t}^{H}+R_{n}\left(t\right)$$

where $R_{\varepsilon }=E\left[\varepsilon \left(t\right)\varepsilon^{H}\left(t\right)\right]=diag\left(\begin{array}{cccc} {\left|\varepsilon_{1}\right|}^{2} & {\left|\varepsilon_{2}\right|}^{2} & \cdots & {\left|\varepsilon_{P}\right|}^{2} \end{array}\right)$, $R_{n}\left(t\right)=E\left[n\left(t\right)n{\left(t\right)}^{H}\right]$.

Assuming that DOD and DOA change slowly, and consider that the DOD and DOA of the target are the same in the time interval $\left[\left(k-1\right)T_{s},kT_{s}\right]$, $\varepsilon_{i}$ and $\varepsilon_{j}$ are uncorrelated for the different targets and all targets are in the same range bin. During the interval $\left[\left(k-1\right)T_{s},kT_{s}\right]$, $\varphi_{t,P},\theta_{t,P}$ remains constant and L snapshots of sensor data are available for the signal processing. 

## 3. Angle Tracking Algorithm Description

The angle tracking algorithm in [19] requires that the steering vector satisfies the Vandermonde form. The joint steering vector in Equation (4) does not satisfy the Vandermonde form, so the angle tracking algorithm in [19] cannot be applied directly to bistatic MIMO radar. We improved the algorithm in [19] and proposed an angle tracking algorithm suitable for bistatic MIMO radar.

### 3.1. Estimation of the Covariance Matrix Difference and the Angle Difference

At the time t, the DOD and DOA of the P targets are recorded as $\gamma_{t}=\left[\varphi_{t,1},\varphi_{t,2},\cdots ,\varphi_{t,P},\theta_{t,1},\theta_{t,2},\cdots ,\theta_{t,P}\right]$. Similarly, the DOD and DOA at time t+1 is recorded as $\gamma_{t+1}=\left[\varphi_{t+1,1},\varphi_{t+1,2},\cdots ,\varphi_{t+1,P},\theta_{t+1,1},\theta_{t+1,2},\cdots ,\theta_{t+1,P}\right]$.

We define

(6) $$\Delta \gamma_{t}=\gamma_{t+1}-\gamma_{t}$$

where $\Delta \gamma_{t}=\left[\Delta \varphi_{t,1},\Delta \varphi_{t,2},\cdots \Delta \varphi_{t,P},\Delta \theta_{t,1},\Delta \theta_{t,2},\cdots \Delta \theta_{t,P}\right]$ is the angle difference between t and t+1, $\Delta \varphi_{t,i}=\varphi_{t+1,i}-\varphi_{t,i}$ and $\Delta \theta_{t,i}=\theta_{t+1,i}-\theta_{t,i}$.

We define $R_{t+1}$ as the covariance matrices of the signal at time t+1. The covariance matrices are

(7) $$R_{t+1}=E\left[y\left(t+1\right)y^{H}\left(t+1\right)\right]=W_{t+1}R_{\varepsilon }W_{t+1}^{H}+R_{n}\left(t+1\right)$$

then we can obtain

(8) $$\Delta R_{t}=R_{t+1}-R_{t}=\left(W_{t+1}R_{\varepsilon }W_{t+1}^{H}-W_{t}R_{\varepsilon }W_{t}^{H}\right)+\left(R_{n}\left(t+1\right)-R_{n}\left(t\right)\right).$$

Supposing that the noise covariance matrix at time t+1 is approximately equal to that at time t, then we have

(9) $$\Delta R_{t}≃W_{t+1}R_{\varepsilon }W_{t+1}^{H}-W_{t}R_{\varepsilon }W_{t}^{H}.$$

It can be seen that the covariance matrix difference of adjacent moment is caused by the angle difference of adjacent moment, so there is a relationship between the two. Therefore, by deriving the relationship between the two, the angle difference of adjacent moment can be obtained.

### 3.2. Estimation of DOD and DOA

In Section 3.1, we obtained the covariance matrix difference $\Delta R_{t}$ and the angle difference $\Delta \gamma_{t}$. This section will deduce the linear relationship between the covariance matrix difference and the angle difference.

We first analyzed the properties of the elements of the covariance matrix. The literature [19] proved that $\Delta R_{t}$ in Equation (9) can be expressed as

(10) $$\Delta R_{t}=\left[\begin{array}{ccccccccc} 0 & b_{1,0} & \cdots & b_{M-1,0} & b_{0,1} & \cdots & b_{M-1,1} & \cdots & b_{M-1,N-1}\\ b_{1,0}^{*} & 0 & \cdots & ∙ & ∙ & ∙ & ∙ & ∙ & ∙\\ \vdots & ∙ & 0 & ∙ & ∙ & ∙ & ∙ & ∙ & ∙\\ b_{M-1,0}^{*} & ∙ & ∙ & 0 & ∙ & ∙ & ∙ & ∙ & ∙\\ b_{0,1}^{*} & ∙ & ∙ & ∙ & 0 & ∙ & ∙ & ∙ & ∙\\ \vdots & ∙ & ∙ & ∙ & ∙ & 0 & ∙ & ∙ & ∙\\ b_{M-1,1}^{*} & ∙ & ∙ & ∙ & ∙ & ∙ & 0 & ∙ & ∙\\ \vdots & ∙ & ∙ & ∙ & ∙ & ∙ & ∙ & 0 & ∙\\ b_{M-1,N-1}^{*} & ∙ & ∙ & ∙ & ∙ & ∙ & ∙ & ∙ & 0 \end{array}\right]$$

where $b_{m,n}=R\left(1,m+n*M+1\right)$, $b_{m,n}^{*}=R\left(m+n*N+1,1\right)$.

(11) $$b_{m,n}=\sum_{i=1}^{P}\rho_{i}\left(e^{-j\pi n\sin\left(\theta_{t,i}+\Delta \theta_{t,i}\right)}e^{-j\pi m\sin\left(\varphi_{t,i}+\Delta \varphi_{t,i}\right)}-e^{-j\pi n\sin\theta_{t,i}}e^{-j\pi m\sin\varphi_{t,i}}\right)$$

where $\rho_{i}$ is the (i,i) element of the matrix $R_{\varepsilon }$ and $\rho_{i}={\left|\varepsilon_{i}\right|}^{2}$, m=0,1,⋯M−1;n=0,1,⋯N−1. $b_{m,n}$ in Equation (11) can be expanded as

(12) $$b_{m,n}=\sum_{i=1}^{P}\rho_{i}\left(e^{-j\pi n\left[\sin\theta_{t,i}\cos\Delta \theta_{t,i}+\cos\theta_{t,i}\sin\Delta \theta_{t,i}\right]}e^{-j\pi m\left[\sin\varphi_{t,i}\cos\Delta \varphi_{t,i}+\cos\varphi_{t,i}\sin\Delta \varphi_{t,i}\right]}-e^{-j\pi n\sin\theta_{t,i}}e^{-j\pi m\sin\varphi_{t,i}}\right).$$

From Equation(12), it can be see that $b_{m,n}$ is related to the angle difference and the angle of the previous moment. $\Delta \theta_{t,i}$ and $\Delta \varphi_{t,i}$ are the parameters to be estimated. Considering that $\Delta \theta_{t,i}$ and $\Delta \varphi_{t,i}$ is very small,

(13) $$\sin\left(\theta_{t,i}+\Delta \theta_{t,i}\right)=\sin\theta_{t,i}\cos\Delta \theta_{t,i}+\cos\theta_{t,i}\sin\Delta \theta_{t,i}≃\sin\theta_{t,i}+\Delta \theta_{t,i}\cos\theta_{t,i}$$

(14) $$\sin\left(\varphi_{t,i}+\Delta \varphi_{t,i}\right)=\sin\varphi_{t,i}\cos\Delta \varphi_{t,i}+\cos\varphi_{t,i}\sin\Delta \varphi_{t,i}≃\sin\varphi_{t,i}+\Delta \varphi_{t,i}\cos\varphi_{t,i}$$

substituting Equations (13) and (14) into Equation(12), then $b_{m,n}$ in Equation (12) can be denoted as

(15) $$b_{m,n}≃\sum_{i=1}^{P}\rho_{i}\left(e^{-j\pi n\left[\sin\theta_{t,i}+\Delta \theta \cos\theta_{t,i}\right]}e^{-j\pi m\left[\sin\varphi_{t,i}+\Delta \varphi_{t,i}\cos\varphi_{t,i}\right]}-e^{-j\pi n\sin\theta_{t,i}}e^{-j\pi m\sin\varphi_{t,i}}\right)=\sum_{i=1}^{P}\rho_{i}\left(e^{-j\pi n\sin\theta_{t,i}}e^{-j\pi m\sin\varphi_{t,i}}\left[e^{-j\pi n\Delta \theta_{t,i}\cos\theta_{t,i}}e^{-j\pi m\Delta \varphi_{t,i}\cos\varphi_{t,i}}-1\right]\right).$$

Considering that x is very small, $e^{x}-1≃x$. Then, $b_{m,n}$ can be rewritten as

(16) $$b_{m,n}\approx \sum_{i=1}^{P}\rho_{i}e^{-j\pi n\sin\theta_{t,i}}e^{-j\pi m\sin\varphi_{t,i}}\left[-j\pi n\Delta \theta_{t,i}\cos\theta_{t,i}-j\pi m\Delta \varphi_{t,i}\cos\varphi_{t,i}\right].$$

By Equation(16), we can construct the following equation

(17) $$V_{t}\Delta \gamma_{t}=b$$

where

(18) $$b={\left[\begin{array}{cccccccccccccccccc} b_{1,0} & b_{2,0} & \cdots & b_{M-1,0} & b_{0,1} & \cdots & b_{M-1,1} & \cdots & b_{M-1,N-1} & b_{1,0}^{*} & b_{2,0}^{*} & \cdots & b_{M-1,0}^{*} & b_{0,1}^{*} & \cdots & b_{M-1}^{*} & \cdots & b_{M-1,N-1}^{*} \end{array}\right]}^{T}$$

(19) $$\Delta \gamma_{t}={\left[\begin{array}{cccccccc} \Delta \varphi_{t,1} & \Delta \varphi_{t,2} & \cdots & \Delta \varphi_{t,P} & \Delta \theta_{t,1} & \Delta \theta_{t,2} & \cdots & \Delta \theta_{t,P} \end{array}\right]}^{T}$$

To give the $V_{t}$, we define $\xi_{\varphi_{i}}=e^{-j\pi \sin\varphi_{i}}$, $\xi_{\theta_{i}}=e^{-j\pi \sin\theta_{i}}$, $\beta_{\varphi_{i}}=-j\pi \cos\varphi_{i}$, $\beta_{\theta_{i}}=-j\pi \cos\theta_{i}$.

(20) $$V_{t}=\left[\begin{array}{cccccc} \rho_{1}\xi_{\varphi_{1}}\beta_{\varphi_{1}} & \cdots & \rho_{P}\xi_{\varphi_{P}}\beta_{\varphi_{P}} & 0 & \cdots & 0\\ \rho_{1}\xi_{\varphi_{1}}^{2}2\beta_{\varphi_{1}} & \cdots & \rho_{P}\xi_{\varphi_{P}}^{2}2\beta_{\varphi_{P}} & 0 & \cdots & 0\\ \vdots & \vdots & \vdots & \vdots & \vdots & \vdots \\ \rho_{1}\xi_{\varphi_{1}}^{M-1}\left(M-1\right)\beta_{\varphi_{1}} & \cdots & \rho_{P}\xi_{\varphi_{P}}^{M-1}\left(M-1\right)\beta_{\varphi_{P}} & 0 & \cdots & 0\\ 0 & \cdots & 0 & \rho_{1}\xi_{\theta_{1}}\beta_{\theta_{1}} & \cdots & \rho_{P}\xi_{\theta_{P}}\beta_{\theta_{P}}\\ \vdots & \vdots & \vdots & \vdots & \vdots & \vdots \\ \rho_{1}\xi_{\varphi_{1}}^{M-1}\xi_{\theta_{1}}\left(M-1\right)\beta_{\varphi_{1}} & \cdots & \rho_{P}\xi_{\varphi_{P}}^{M-1}\xi_{\theta_{P}}\left(M-1\right)\beta_{\varphi_{P}} & \rho_{1}\xi_{\varphi_{1}}^{M-1}\xi_{\theta_{1}}\beta_{\theta_{1}} & \cdots & \rho_{P}\xi_{\varphi_{P}}^{M-1}\xi_{\theta_{P}}\beta_{\theta_{P}}\\ \vdots & \vdots & \vdots & \vdots & \vdots & \vdots \\ 0 & \cdots & 0 & \rho_{1}\xi_{\theta_{1}}^{N-1}\left(N-1\right)\beta_{\theta_{1}} & \cdots & \rho_{P}\xi_{\theta_{P}}^{N-1}\left(N-1\right)\beta_{\theta_{P}}\\ \vdots & \vdots & \vdots & \vdots & \vdots & \vdots \\ \rho_{1}\xi_{\varphi_{1}}^{M-1}\xi_{\theta_{1}}^{N-1}\left(M-1\right)\beta_{\varphi_{1}} & \cdots & \rho_{P}\xi_{\varphi_{P}}^{M-1}\xi_{\theta_{P}}^{N-1}\left(M-1\right)\beta_{\varphi_{P}} & \rho_{1}\xi_{\varphi_{1}}^{M-1}\xi_{\theta_{1}}^{N-1}\left(N-1\right)\beta_{\theta_{1}} & \cdots & \rho_{P}\xi_{\varphi_{P}}^{M-1}\xi_{\theta_{P}}^{N-1}\left(N-1\right)\beta_{\theta_{P}}\\ \rho_{1}\xi_{\varphi_{1}}^{-1}\left(-\beta_{\varphi_{1}}\right) & \cdots & \rho_{P}\xi_{\varphi_{P}}^{-1}\left(-\beta_{\varphi_{P}}\right) & 0 & \cdots & 0\\ \rho_{1}\xi_{\varphi_{1}}^{-2}(-2)\beta_{\varphi_{1}} & \cdots & \rho_{P}\xi_{\varphi_{P}}^{-2}(-2)\beta_{\varphi_{P}} & 0 & \cdots & 0\\ \vdots & \vdots & \vdots & \vdots & \vdots & \vdots \\ \rho_{1}\xi_{\varphi_{1}}^{-\left(M-1\right)}\left(-\left(M-1\right)\right)\beta_{\varphi_{1}} & \cdots & \rho_{P}\xi_{\varphi_{P}}^{-\left(M-1\right)}\left(-\left(M-1\right)\right)\beta_{\varphi_{P}} & 0 & \cdots & 0\\ 0 & \cdots & 0 & \rho_{1}\xi_{\theta_{1}}\beta_{\theta_{1}} & \cdots & \rho_{P}\xi_{\theta_{P}}\beta_{\theta_{P}}\\ \vdots & \vdots & \vdots & \vdots & \vdots & \vdots \\ \rho_{1}\xi_{\varphi_{1}}^{-\left(M-1\right)}\xi_{\theta_{1}}^{-1}\left(-\left(M-1\right)\right)\beta_{\varphi_{1}} & \cdots & \rho_{P}\xi_{\varphi_{P}}^{-\left(M-1\right)}\xi_{\theta_{P}}^{-1}\left(-\left(M-1\right)\right)\beta_{\varphi_{P}} & \rho_{1}\xi_{\varphi_{1}}^{-\left(M-1\right)}\xi_{\theta_{1}}^{-1}\beta_{\theta_{1}} & \cdots & \rho_{P}\xi_{\varphi_{P}}^{-\left(M-1\right)}\xi_{\theta_{P}}^{-1}\beta_{\theta_{P}}\\ \vdots & \vdots & \vdots & \vdots & \vdots & \vdots \\ 0 & \cdots & 0 & \rho_{1}\xi_{\theta_{1}}^{-\left(N-1\right)}\left(-\left(N-1\right)\right)\beta_{\theta_{1}} & \cdots & \rho_{P}\xi_{\theta_{P}}^{-\left(N-1\right)}\left(-\left(N-1\right)\right)\beta_{\theta_{P}}\\ \vdots & \vdots & \vdots & \vdots & \vdots & \vdots \\ \rho_{1}\xi_{\varphi_{1}}^{-\left(M-1\right)}\xi_{\theta_{1}}^{-\left(N-1\right)}\left(-\left(M-1\right)\right)\beta_{\varphi_{1}} & \cdots & \rho_{P}\xi_{\varphi_{P}}^{-\left(M-1\right)}\xi_{\theta_{P}}^{-\left(N-1\right)}\left(-\left(M-1\right)\right)\beta_{\varphi_{P}} & \rho_{1}\xi_{\varphi_{1}}^{-\left(M-1\right)}\xi_{\theta_{1}}^{-\left(N-1\right)}\left(-\left(N-1\right)\right)\beta_{\theta_{1}} & \cdots & \rho_{P}\xi_{\varphi_{P}}^{-\left(M-1\right)}\xi_{\theta_{P}}^{-\left(N-1\right)}\left(-\left(N-1\right)\right)\beta_{\theta_{P}} \end{array}\right]$$

Using the least square method to estimate $\Delta \gamma_{t}$ in Equation (17), we get

(21) $$\Delta \gamma_{t}={\left(V_{t}^{H}V_{t}\right)}^{-1}V_{t}^{H}b.$$

The final estimate of the angle is

(22) $$\gamma_{t+1}=\gamma_{t}+\Delta \gamma_{t}$$

### 3.3. Covariance Element Average Operation

The algorithm in [19] makes full use of the Toeplitz matrix property to improve performance. Since the joint steering vector of bistatic MIMO radar does not satisfy the Vandermonde form, the covariance matrix difference does not satisfy the Toeplitz property. Therefore, we first analyzed the structure of the steering vector.

(23) Wt(φ,θ)=[1,ejπsinφt,i⋯,ejπ(M−1)sinφt,i,ejπsinθt,i,ejπsinθt,iejπsinφt,i,⋯,ejπsinθt,iejπ(M−1)sinφt,i,⋯,        ejπ(N−1)sinθt,i,ejπ(N−1)sinθt,iejπsinφt,i,⋯,ejπ(N−1)sinθt,iejπ(M−1)sinφt,i]T.

It can be found that some elements in the steering vector satisfied the Vandermonde structure in Equation (23). This structure is called the approximate Vandermonde structure in this paper.

We further analyzed the structure of the covariance matrix, taking $R_{t}$ as an example.

(24) $$R_{t}=\left[\begin{array}{ccccccccccccc} r_{1,1} & r_{1,2} & \cdots & r_{1,M} & r_{1,M+1} & r_{1,M+2} & \cdots & r_{1,2M} & \cdots & r_{1,\left(N-1\right)M+1} & r_{1,\left(N-1\right)M+2} & \cdots & r_{1,NM}\\ r_{1,2}^{*} & r_{1,1} & \cdots & r_{1,M-1} & & & & & & & & & \\ \vdots & \vdots & \ddots & \vdots & & & & & & & & & \\ r_{1,M}^{*} & r_{1,M-1}^{*} & \cdots & r_{1,1} & & & & & & & & & \\ r_{1,M+1}^{*} & & & & r_{1,1} & r_{1,2} & \cdots & r_{1,M} & & & & & \\ r_{1,M+2}^{*} & & & & r_{1,2}^{*} & r_{1,1} & \cdots & r_{1,M-1} & & & & & \\ \vdots & & & & \vdots & \vdots & \ddots & \vdots & & & & & \\ r_{1,2M}^{*} & & & & r_{1,M}^{*} & r_{1,M-1}^{*} & \cdots & r_{1,1} & & & & & \\ \vdots & & & & & & & & \ddots & & & & \\ r_{1,\left(N-1\right)M+1}^{*} & & & & & & & & & r_{1,1} & r_{1,2} & \cdots & r_{1,M}\\ r_{1,\left(N-1\right)M+2}^{*} & & & & & & & & & r_{1,2}^{*} & r_{1,1} & \cdots & r_{1,M-1}\\ \vdots & & & & & & & & & \vdots & \vdots & r_{1,1} & \vdots \\ r_{1,NM}^{*} & & & & & & & & & r_{1,M}^{*} & r_{1,M-1}^{*} & \cdots & r_{1,1} \end{array}\right]$$

Since the steering vector satisfied the approximate Vandermonde structure, the sub-matrices in a straight line parallel to the principal diagonal of $R_{t}$ are the same. $R_{t+1}$ has a similar structure.

From the above analysis, we can see that $\Delta R_{t}$ is an approximate Toeplitz matrix whose sub-matrices in a straight line parallel to the principal diagonal are the same. We used the average operation to estimate $b_{m,n}$ and $b_{m,n}^{*}$ to eliminate the noise. The following steps can be used to update the $b_{m,n}$ and $b_{m,n}^{*}$.

(25) $$\begin{array}{l} {\hat{b}}_{m,n}=\frac{1}{\left(M-m\right)\left(N-n\right)}\sum_{k=1}^{M-m}\sum_{kk=n+1}^{N}\Delta R_{t}\left(k+\left(kk-n-1\right)M,k+m+\left(kk-1\right)M\right)\\ {\hat{b}}_{m,n}^{*}=\frac{1}{\left(M-m\right)\left(N-n\right)}\sum_{k=1}^{M-m}\sum_{kk=n+1}^{N}\Delta R_{t}\left(k+m+\left(kk-1\right)M,k+\left(kk-n-1\right)M\right) \end{array}$$

where $\Delta R_{t}\left(i,j\right)$ is the (i,j) element of the matrix $\Delta R_{t}$. Substituting Equation (25) into Equation (18), then b can be rewritten as

(26) $$b={\left[\begin{array}{cccccccccccccccccc} {\hat{b}}_{1,0} & {\hat{b}}_{2,0} & \cdots & {\hat{b}}_{M-1,0} & {\hat{b}}_{0,1} & \cdots & {\hat{b}}_{M-1,1} & \cdots & {\hat{b}}_{M-1,N-1} & {\hat{b}}_{1,0}^{*} & {\hat{b}}_{2,0}^{*} & \cdots & {\hat{b}}_{M-1,0}^{*} & {\hat{b}}_{0,1}^{*} & \cdots & {\hat{b}}_{M-1}^{*} & \cdots & {\hat{b}}_{M-1,N-1}^{*} \end{array}\right]}^{T}$$

The proposed algorithm fully uses the approximate Toeplitz matrix property and more receiving information to eliminate the noise, and improve angle tracking performance. Now, we discuss the performance comparison between our algorithm and the algorithms in the literature [19,20,21,22].

The AAJD and PASTd algorithm sestimate the target angle through optimizing the function. Because it is difficult to find the optimal solution of the optimization function, the performance of the algorithm is low. The algorithm in [19,21] and our algorithm obtain angle via the difference between the previous and current covariance matrix of the receiving signal. So the performance of the algorithm in [19,21] and our algorithm is better than the AAJD and PASTd algorithms.

The algorithm in [19] can be improved to solve the angle tracking problem of bistatic MIMO radar, but it only uses the $\left(M^{2}+M\right)\left(N-1\right)+M^{2}-M$ elements of the covariance matrix (M is the number of transmit antennas, and N is the number of receive antennas). The algorithm in [21] and our algorithm uses the 2(MN−1) and $\frac{\left(M^{2}+M\right)\left(N^{2}-N\right)}{2}+\left(M^{2}-M\right)N$ elements of the covariance matrix to track the target, respectively. Owning to $\frac{\left(M^{2}+M\right)\left(N^{2}-N\right)}{2}+\left(M^{2}-M\right)N>\left(M^{2}+M\right)\left(N-1\right)+M^{2}-M>2\left(MN-1\right)$, the proposed algorithm used more covariance matrix information than the algorithm in [19,21]. So, the performance of our algorithm was better than that of [19,21].

In summary, the performance of our algorithm was the best.

Until now, we show the major steps of the angle tracking algorithm in bistatic MIMO radar as follows.

Step 1. Calculate the covariance matrix $R_{t}$ and $R_{t+1}$ via Equations (5) and (7).

Step 2. Calculate the covariance matrix difference $\Delta R_{t}$ via Equation (10).

Step 3. The vector b is obtained via Equation (26), and the vector $V_{t}$ is obtained via Equation (20).

Step 4. We estimate $\Delta \gamma_{t}$ via Equation (21), and the angle at time t+1 is $\gamma_{t+1}=\gamma_{t}+\Delta \gamma_{t}=\gamma_{1}+\sum_{i=1}^{t}\Delta \gamma_{i}$.

Step 5. Repeat steps 1 to 4 to estimate the angle of the next moment.

Note 1. This paper assumes that the number of targets in the bistatic MIMO radar is known. If we do not know the number in advance, we can use the existing target-number estimation algorithm in [23] to estimate the number of targets.
Note 2. The algorithm in this paper only obtains the angle difference of adjacent time, and therefore we need to get the initial DOD and DOA of the target. The initial DOD and DOA can be obtained using the MUSIC algorithm or another angle estimation algorithm.
Note 3. The algorithm in this paper was valid effectively when the target velocity was low and the DOD and DOA changed slowly. When the target moves faster, the performance of the algorithm in this paper will be reduced or even invalidated. When the target was far from the transceiver base, the angle difference was generally small, so this algorithm is suitable for tracking the long-distance target.
Note 4. The noise covariance matrices of adjacent time can be approximately assumed to be equal. No matter what kind of noise, the noise component in Equation (8) can be eliminated. The algorithm is still effective under the colored noise conditions.

### 3.4. Computational Complexity Analysis and Advantages of the Proposed Algorithm

For the proposed algorithm, the calculation of the covariance matrix needs $O\left({\left(MN\right)}^{2}L\right)$, and the computation of ${\left(V_{t}^{H}V_{t}\right)}^{-1}V_{t}^{H}b$ requires $O\left(2P^{2}\left(MN-1\right)+P^{3}+2P\left(MN-1\right)+P^{2}\right)$. The main computational complexity of the proposed algorithm is $O\left({\left(MN\right)}^{2}L+2P^{2}\left(MN-1\right)+P^{3}+2P\left(MN-1\right)+P^{2}\right)$.

The advantages of this algorithm are listed as follows:

(1) The proposed algorithm does not need eigenvalue decomposition of the covariance matrix, so the complexity is lower.
(2) The proposed algorithm not only introduces the tracking algorithm in [19], but also expands it.
(3) The proposed algorithm makes full use of the elements in the covariance matrix to improve the tracking performance. The performance of this algorithm is better than the AAJD algorithm.
(4) The algorithm in this paper can automatically match and associate the angles of adjacent moment and reduce the computational complexity.

## 4. Error Analysis

In this section, we deduce the variance of DOD and DOA tracking. We assume that the observed noise variances are nearly the same at the adjacent time. When estimating DOD and DOA, we used approximate calculations such as $e^{x}-1\approx x$ and sinx≈x when x was smaller. This leads to a slight difference from the real value. Consider

(27) $$\sin x=x-\frac{x^{3}}{3!}+\Lambda$$

(28) $$\cos x=1-\frac{x^{2}}{2!}+\Lambda^{\prime }$$

(29) $$e^{x}-1=x+\Lambda^{\prime \prime }$$

where Λ, $\Lambda^{\prime }$, and $\Lambda^{\prime \prime }$ are the high-order expansion terms.

According to Equations (13), (14), (27) and (28), we have

(30) $$\begin{array}{l} b_{m,n}=\sum_{i=1}^{P}\rho_{i}(e^{-j\pi n\left[\sin\theta_{t,i}\cos\Delta \theta_{t,i}+\cos\theta_{t,i}\sin\Delta \theta_{t,i}\right]}e^{-j\pi m\left[\sin\varphi_{t,i}\cos\Delta \varphi_{t,i}+\cos\varphi_{t,i}\sin\Delta \varphi_{t,i}\right]}-e^{-j\pi n\sin\theta_{t,i}}e^{-j\pi m\sin\varphi_{t,i}})\\ =\sum_{i=1}^{P}\rho_{i}(e^{-j\pi n\left[\sin\theta_{t,i}\left(1-\left(\Delta \theta_{t,i}^{2}/2!\right)+\Lambda^{\prime }\right)+\cos\theta_{t,i}\left(\Delta \theta_{t,i}-\left(\Delta \theta_{t,i}^{3}/3!\right)+\Lambda \right)\right]}e^{-j\pi m\left[\sin\varphi_{t,i}\left(1-\left(\Delta \varphi_{t,i}^{2}/2!\right)+\Lambda^{\prime }\right)+\cos\varphi_{t,i}\left(\Delta \varphi_{t,i}-\left(\Delta \varphi_{t,i}^{3}/3!\right)+\Lambda \right)\right]}-e^{-j\pi n\sin\theta_{t,i}}e^{-j\pi m\sin\varphi_{t,i}})\\ =\sum_{i=1}^{P}\rho_{i}(e^{-j\pi n\left[\sin\theta_{t,i}+\cos\theta_{t,i}\Delta \theta_{t,i}\right]}e^{-j\pi n\left[\sin\theta_{t,i}\left(-\left(\Delta \theta_{t,i}^{2}/2!\right)+\Lambda^{\prime }\right)+\cos\varphi_{t,i}\left(-\left(\Delta \theta_{t,i}^{3}/3!\right)+\Lambda \right)\right]}e^{-j\pi m\left[\sin\varphi_{t,i}+\cos\varphi_{t,i}\Delta \varphi_{t,i}\right]}e^{-j\pi m\left[\sin\varphi_{t,i}\left(-\left(\Delta \varphi_{t,i}^{2}/2!\right)+\Lambda^{\prime }\right)+\cos\varphi_{t,i}\left(-\left(\Delta \varphi_{t,i}^{3}/3!\right)+\Lambda \right)\right]}-e^{-j\pi n\sin\theta_{t,i}}e^{-j\pi m\sin\varphi_{t,i}})\\ =\sum_{i=1}^{P}\rho_{i}e^{-j\pi \left(n\sin\theta_{t,i}+m\sin\varphi_{t,i}\right)}(e^{-j\pi \left(n\cos\theta_{t,i}\Delta \theta_{t,i}+m\cos\varphi_{t,i}\Delta \varphi_{t,i}\right)}e^{-j\pi n\left[\sin\theta_{t,i}\left(-\left(\Delta \theta_{t,i}^{2}/2!\right)+\Lambda^{\prime }\right)+\cos\theta_{t,i}\left(-\left(\Delta \theta_{t,i}^{3}/3!\right)+\Lambda \right)\right]}e^{-j\pi m\left[\sin\varphi_{t,i}\left(-\left(\Delta \varphi_{t,i}^{2}/2!\right)+\Lambda^{\prime }\right)+\cos\varphi_{t,i}\left(-\left(\Delta \varphi_{t,i}^{3}/3!\right)+\Lambda \right)\right]}-1)\\ =\sum_{i=1}^{P}\rho_{i}e^{-j\pi \left(n\sin\theta_{t,i}+m\sin\varphi_{t,i}\right)}(e^{-j\pi \left(n\cos\theta_{t,i}\Delta \theta_{t,i}+m\cos\varphi_{t,i}\Delta \varphi_{t,i}\right)-j\pi n\left[\sin\theta_{t,i}\left(-\left(\Delta \theta_{t,i}^{2}/2!\right)+\Lambda^{\prime }\right)+\cos\theta_{t,i}\left(-\left(\Delta \theta_{t,i}^{3}/3!\right)+\Lambda \right)\right]-j\pi m\left[\sin\varphi_{t,i}\left(-\left(\Delta \varphi_{t,i}^{2}/2!\right)+\Lambda^{\prime }\right)+\cos\varphi_{t,i}\left(-\left(\Delta \varphi_{t,i}^{3}/3!\right)+\Lambda \right)\right]}-1) \end{array}$$

By Equations (15), (16), and (29), then

(31) $$\begin{array}{l} b_{m,n}=\sum_{i=1}^{P}\rho_{i}e^{-j\pi \left(n\sin\theta_{t,i}+m\sin\varphi_{t,i}\right)}(-j\pi \left(n\cos\theta_{t,i}\Delta \theta_{t,i}+m\cos\varphi_{t,i}\Delta \varphi_{t,i}\right)-j\pi n\left[\sin\theta_{t,i}\left(-\left(\Delta \theta_{t,i}^{2}/2!\right)+\Lambda^{\prime }\right)+\cos\theta_{t,i}\left(-\left(\Delta \theta_{t,i}^{3}/3!\right)+\Lambda \right)\right]\\ -j\pi m\left[\sin\varphi_{t,i}\left(-\left(\Delta \varphi_{t,i}^{2}/2!\right)+\Lambda^{\prime }\right)+\cos\varphi_{t,i}\left(-\left(\Delta \varphi_{t,i}^{3}/3!\right)+\Lambda \right)\right]+j\Lambda^{\prime \prime })\\ =-\sum_{i=1}^{P}\rho_{i}e^{-j\pi \left(n\sin\theta_{t,i}+m\sin\varphi_{t,i}\right)}j\pi \left(n\cos\theta_{t,i}\Delta \theta_{t,i}+m\cos\varphi_{t,i}\Delta \varphi_{t,i}\right)+\sum_{i=1}^{P}\rho_{i}e^{-j\pi \left(n\sin\theta_{t,i}+m\sin\varphi_{t,i}\right)}(-j\pi n\left[\sin\theta_{t,i}\left(-\left(\Delta \theta_{t,i}^{2}/2!\right)+\Lambda^{\prime }\right)+\cos\theta_{t,i}\left(-\left(\Delta \theta_{t,i}^{3}/3!\right)+\Lambda \right)\right]\\ -j\pi m\left[\sin\varphi_{t,i}\left(-\left(\Delta \varphi_{t,i}^{2}/2!\right)+\Lambda^{\prime }\right)+\cos\varphi_{t,i}\left(-\left(\Delta \varphi_{t,i}^{3}/3!\right)+\Lambda \right)\right]+j\Lambda^{\prime \prime }) \end{array}$$

$\partial b_{m,n}$ is the estimation error of $b_{m,n}$, and $\partial b_{m,n}$ can be shown as follows:

(32) $$\begin{array}{r} \partial b_{m,n}=\sum_{i=1}^{P}\rho_{i}e^{-j\pi \left(n\sin\theta_{t,i}+m\sin\varphi_{t,i}\right)}(-j\pi n\left[\sin\theta_{t,i}\left(-\left(\Delta \theta_{t,i}^{2}/2!\right)+\Lambda^{\prime }\right)+\cos\theta_{t,i}\left(-\left(\Delta \theta_{t,i}^{3}/3!\right)+\Lambda \right)\right]\\ -j\pi m\left[\sin\varphi_{t,i}\left(-\left(\Delta \varphi_{t,i}^{2}/2!\right)+\Lambda^{\prime }\right)+\cos\varphi_{t,i}\left(-\left(\Delta \varphi_{t,i}^{3}/3!\right)+\Lambda \right)\right]+j\Lambda^{\prime \prime }) \end{array}$$

According to Equations (21) and (32), the variance of $\Delta b_{m,n}$ is denoted by

(33) $$\begin{array}{l} E\left[{\left|\partial b_{m,n}\right|}^{2}\right]=\frac{1}{\left(M-m\right)\left(N-n\right)}\sum_{i=1}^{P}\rho_{i}^{2}(\pi n\left[\sin\theta_{t,i}\left(-\left(\Delta \theta_{t,i}^{2}/2!\right)+\Lambda^{\prime }\right)+\cos\theta_{t,i}\left(-\left(\Delta \theta_{t,i}^{3}/3!\right)+\Lambda \right)\right]\\ \;{+\pi m\left[\sin\varphi_{t,i}\left(-\left(\Delta \varphi_{t,i}^{2}/2!\right)+\Lambda^{\prime }\right)+\cos\varphi_{t,i}\left(-\left(\Delta \varphi_{t,i}^{3}/3!\right)+\Lambda \right)\right]+\Lambda^{\prime \prime })}^{2} \end{array}$$

Then, we have

(34) $$E\left[\partial b_{m,n}\partial b_{r,s}^{*}\right]=0,\forall m\neq r,n\neq s$$

(35) $$E\left[\partial b_{m,n}^{2}\right]=0$$

We define that ∂b is the estimation error of b.

(36) $$\partial b=\begin{array}{cccccc} {}[\partial {\hat{b}}_{1,0} & \partial {\hat{b}}_{2,0} & \cdots & \partial {\hat{b}}_{M-1,0} & \partial {\hat{b}}_{0,1} & \cdots \end{array}{\begin{array}{ccccccc} \partial {\hat{b}}_{M-1,1} & \cdots & \partial {\hat{b}}_{M-1,N-1} & \partial {\hat{b}}_{1,0}^{*} & \partial {\hat{b}}_{2,0}^{*} & \cdots & \partial {\hat{b}}_{M-1,N-1}^{*} \end{array}]}^{T}$$

Then the variance of ∂b is

(37) $$E\left[{\left|\partial b\right|}^{2}\right]=\left[\begin{array}{c} E\left[{\left|\partial b_{1,0}\right|}^{2}\right]\\ \vdots \\ E\left[{\left|\partial b_{M-1,0}\right|}^{2}\right]\\ E\left[{\left|\partial b_{0,1}\right|}^{2}\right]\\ \vdots \\ E\left[{\left|\partial b_{M-1,1}\right|}^{2}\right]\\ \vdots \\ E\left[{\left|\partial b_{M-1,N-1}\right|}^{2}\right]\\ E\left[{\left|\partial b_{1,0}\right|}^{2}\right]\\ \vdots \\ E\left[{\left|\partial b_{M-1,N-1}\right|}^{2}\right] \end{array}\right]=\left[\begin{array}{c} \frac{1}{\left(M-1\right)N}\sum_{i=1}^{P}\rho_{i}^{2}{\left(\pi \left[\sin\varphi_{t,i}\left(-\left(\Delta \varphi_{t,i}^{2}/2!\right)+\Lambda^{\prime }\right)+\cos\varphi_{t,i}\left(-\left(\Delta \varphi_{t,i}^{3}/3!\right)+\Lambda \right)\right]+\Lambda^{\prime \prime }\right)}^{2}\\ \vdots \\ \frac{1}{N}\sum_{i=1}^{P}\rho_{i}^{2}{\left(\pi \left(M-1\right)\left[\sin\varphi_{t,i}\left(-\left(\Delta \varphi_{t,i}^{2}/2!\right)+\Lambda^{\prime }\right)+\cos\varphi_{t,i}\left(-\left(\Delta \varphi_{t,i}^{3}/3!\right)+\Lambda \right)\right]+\Lambda^{\prime \prime }\right)}^{2}\\ \frac{1}{M\left(N-1\right)}\sum_{i=1}^{P}\rho_{i}^{2}{\left(\pi \left[\sin\theta_{t,i}\left(-\left(\Delta \theta_{t,i}^{2}/2!\right)+\Lambda^{\prime }\right)+\cos\theta_{t,i}\left(-\left(\Delta \theta_{t,i}^{3}/3!\right)+\Lambda \right)\right]+\Lambda^{\prime \prime }\right)}^{2}\\ \vdots \\ \begin{array}{l} \frac{1}{\left(N-1\right)}\sum_{i=1}^{P}\rho_{i}^{2}(\pi \left[\sin\theta_{t,i}\left(-\left(\Delta \theta_{t,i}^{2}/2!\right)+\Lambda^{\prime }\right)+\cos\theta_{t,i}\left(-\left(\Delta \theta_{t,i}^{3}/3!\right)+\Lambda \right)\right]+\\ {\pi \left(M-1\right)\left[\sin\varphi_{t,i}\left(-\left(\Delta \varphi_{t,i}^{2}/2!\right)+\Lambda^{\prime }\right)+\cos\varphi_{t,i}\left(-\left(\Delta \varphi_{t,i}^{3}/3!\right)+\Lambda \right)\right]+\Lambda^{\prime \prime })}^{2} \end{array}\\ \vdots \\ \begin{array}{l} \sum_{i=1}^{P}\rho_{i}^{2}(\pi \left(N-1\right)\left[\sin\theta_{t,i}\left(-\left(\Delta \theta_{t,i}^{2}/2!\right)+\Lambda^{\prime }\right)+\cos\theta_{t,i}\left(-\left(\Delta \theta_{t,i}^{3}/3!\right)+\Lambda \right)\right]+\\ {\pi \left(M-1\right)\left[\sin\varphi_{t,i}\left(-\left(\Delta \varphi_{t,i}^{2}/2!\right)+\Lambda^{\prime }\right)+\cos\varphi_{t,i}\left(-\left(\Delta \varphi_{t,i}^{3}/3!\right)+\Lambda \right)\right]+\Lambda^{\prime \prime })}^{2} \end{array}\\ \frac{1}{\left(M-1\right)N}\sum_{i=1}^{P}\rho_{i}^{2}{\left(\pi \left[\sin\varphi_{t,i}\left(-\left(\Delta \varphi_{t,i}^{2}/2!\right)+\Lambda^{\prime }\right)+\cos\varphi_{t,i}\left(-\left(\Delta \varphi_{t,i}^{3}/3!\right)+\Lambda \right)\right]+\Lambda^{\prime \prime }\right)}^{2}\\ \vdots \\ \begin{array}{l} \sum_{i=1}^{P}\rho_{i}^{2}(\pi \left(N-1\right)\left[\sin\theta_{t,i}\left(-\left(\Delta \theta_{t,i}^{2}/2!\right)+\Lambda^{\prime }\right)+\cos\theta_{t,i}\left(-\left(\Delta \theta_{t,i}^{3}/3!\right)+\Lambda \right)\right]+\\ {\pi \left(M-1\right)\left[\sin\varphi_{t,i}\left(-\left(\Delta \varphi_{t,i}^{2}/2!\right)+\Lambda^{\prime }\right)+\cos\varphi_{t,i}\left(-\left(\Delta \varphi_{t,i}^{3}/3!\right)+\Lambda \right)\right]+\Lambda^{\prime \prime })}^{2} \end{array} \end{array}\right]$$

According to [24], we get the variance of $\Delta \gamma_{t,i}$

(38) var[Δγt,i]=E[Vt,i+∂b]+Re(E[Vt,i+∂b]2)2

where $V_{t,i}^{+}$ denotes the ith row of $V_{t}^{+}$ and $V_{t}^{+}$ is the pseudoinverse of $V_{t}$. According to Equations (33)–(35) and (37), we get

(39) var[Δγt,i]=Vt,i+diag(E[|∂b|2]Vt,i+H+Re(Vt,i+E[∂b∂bT]Vt,i+T))2=Vt,i+diag(E[|∂b|2])Vt,i+H2

where $E\left[{\left|\partial b\right|}^{2}\right]$ is shown in Equation (37).

From Equations (37) and (39), we can obtain an effective conclusion where the theoretical variance of the proposed algorithm is gradually decreased with the number of transmit/receive antennas increases. Multiple transmit/receive antennas improve the angle tracking performance.

## 5. Simulation Results

Assuming that both the transmit and receive arrays of the bistatic MIMO radar are linearly configured, the spacing of the array elements is half wavelength. The carrier frequency of the array element is 1 GHz, the pulse width is 10 μs, and the pulse repetition rate is 10 kHz. The emission waveform uses the Hadamard Code Pulse (HCP) signal, and the number of the transmit and receive array elements is M=N=5 (except experiments 6,7). We defined the root-mean square error (RMSE) as $RMSE\left(\theta \right)=\sqrt{\frac{1}{F}\sum_{m=1}^{F}\frac{1}{P}\sum_{k=1}^{P}\frac{1}{T}\sum_{t=1}^{T}\left[{\left({\hat{\theta }}_{k,m,t}-\theta_{k,m,t}\right)}^{2}+{\left({\hat{\varphi }}_{k,m,t}-\varphi_{k,m,t}\right)}^{2}\right]}$, where ${\hat{\theta }}_{k,m,t}$ and ${\hat{\varphi }}_{k,m,t}$ is the estimate of angle $\theta_{k,m,t}$ and $\varphi_{k,m,t}$; F are the times of the Monte Carlo trial. The targets are tracked over an interval of 6 s, during each 0.1 s interval, L snapshots of sensor data are generated and used to estimate angles.

**Figure 2:**
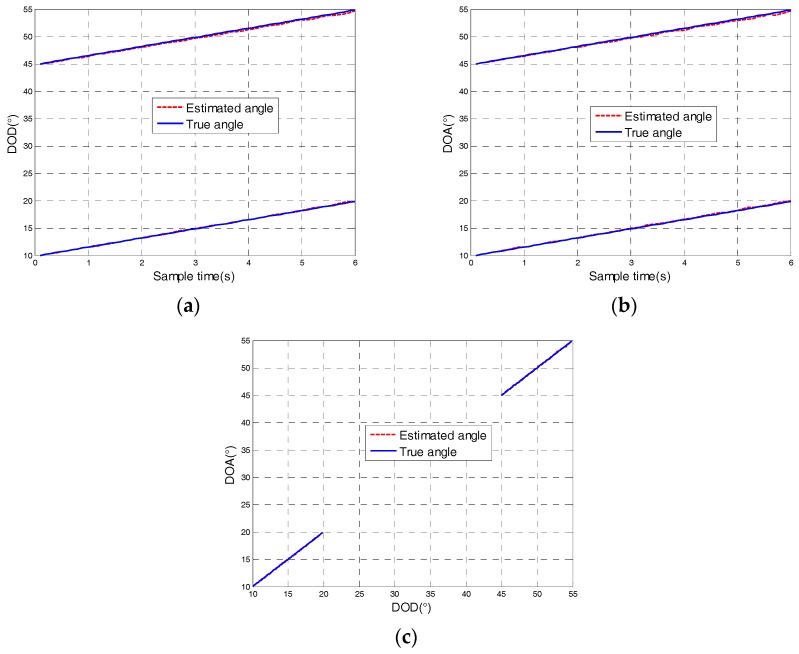
Angle tracking results of uniform moving target at SNR=15 dB: (**a**) The direction of departure (DOD); (**b**) The direction of arrival (DOA); (**c**) DOD and DOA trajectory.

Figure 2 shows the result of the tracking angle of the targets of uniform speed for P=2, L=100, and SNR=15 dB. The simulation results showed that the estimated trajectory coincided with the real trajectory, which proved the effectiveness of the algorithm.

**Figure 3:**
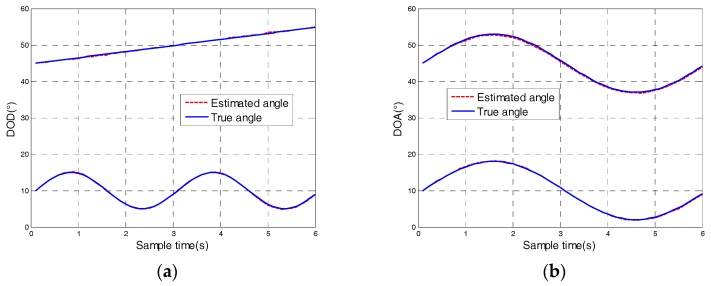

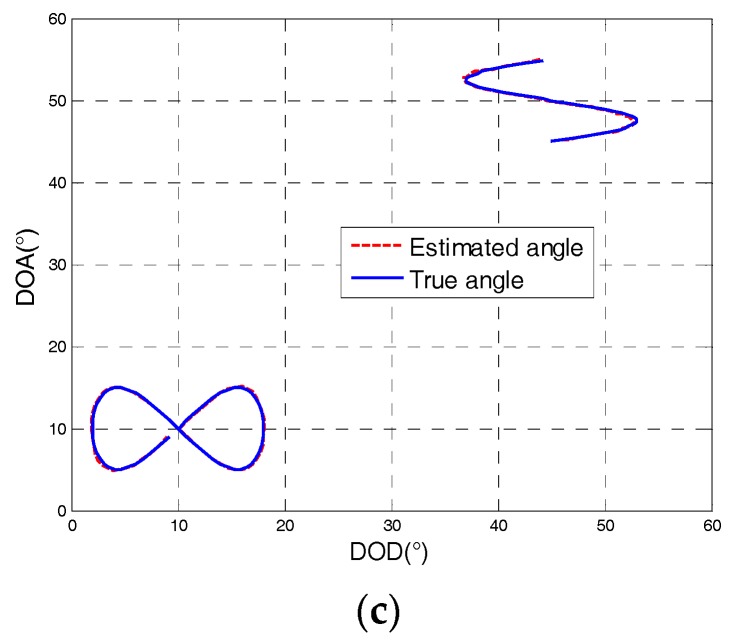
Angle tracking results of non-uniform moving targets with SNR=15 dB: (**a**) The direction of departure; (**b**) The direction of arrival; (**c**) DOD and DOA trajectory.

Figure 3 depicts the tracking result of the proposed algorithm for non-uniform moving targets and showed that our algorithm could successfully track the moving target at a non-uniform speed. The DOD and DOA could be automatically associated. The estimated angle trajectory coincided with the true trajectory, indicating the effectiveness and robustness of the proposed algorithm.

**Figure 4:**
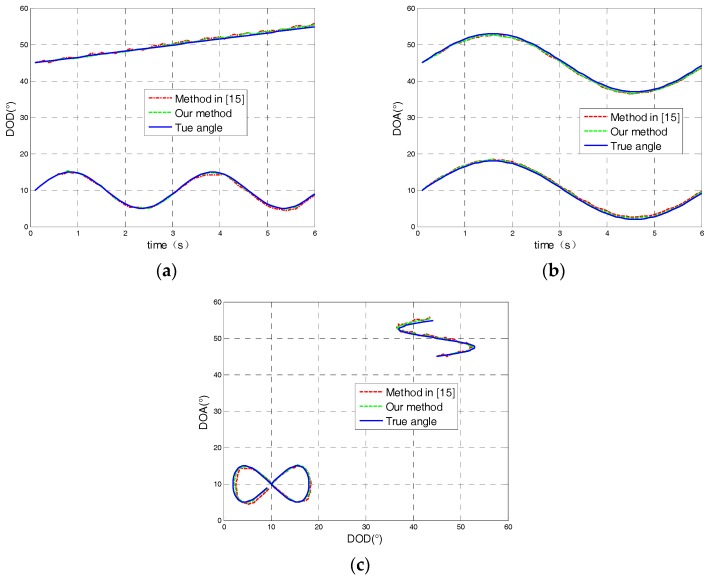
Angle tracking result comparison with SNR=10 dB: (**a**) The direction of departure; (**b**) The direction of arrival; (**c**) DOD and DOA trajectory comparison.

Figure 4 depicts the tracking result comparison between the proposed algorithm and the angle tracking algorithm in [19] with SNR=10 dB. From Figure 4, it can be seen that the tracking result of our algorithm had a high degree of coincidence with the real trajectory of the target, and the performance was better than the tracking algorithm in [19].

**Figure 5:**
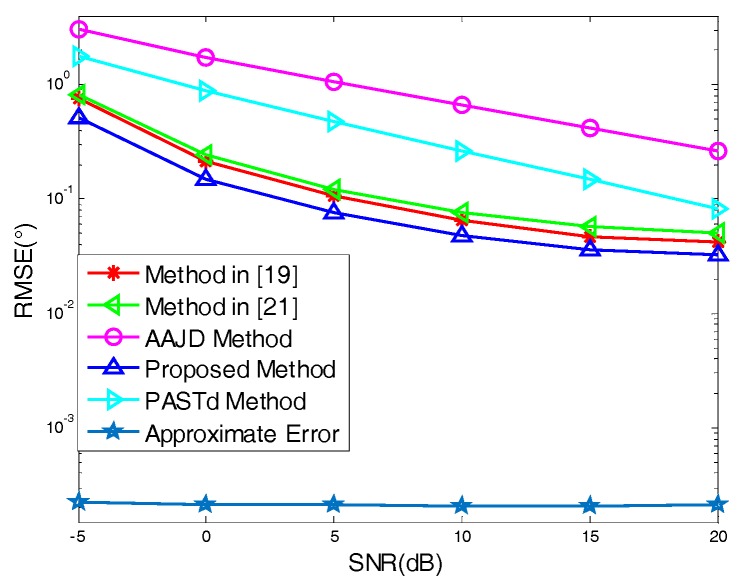
RMSE changes with signal-to-noise ratio (SNR).

To better verify the performance of the proposed algorithm, Figure 5 shows the tracking performance comparison with P=2, L=100, F=200, and SNR=−5–10 dB, where we compared the proposed algorithm against the angle tracking algorithm in [19,21], the PASTd algorithm in [20], and AAJD algorithm in [22]. At the same time, we gave the theoretical error caused by the approximate operation. It can be seen from Figure 5 that the RMSE of our algorithm was lower than that of the angle tracking algorithm in [19,21], PASTd algorithm, and AAJD algorithm, which showed that the tracking performance of our algorithm was the best and the correctness of the theoretical analysis was verified. This was because the proposed algorithm made full use of the elements of covariance matrix to eliminate the noise and improved the estimation performance. The theoretical variance was lower than the actual variance, because the noise was ignored when the theoretical variance is derived.

**Figure 6:**
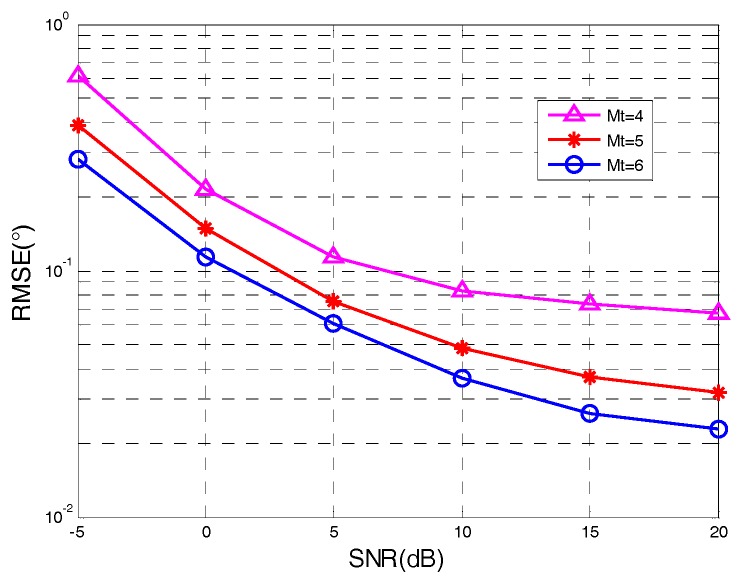
Angle tracking with different values of Mt.

**Figure 7:**
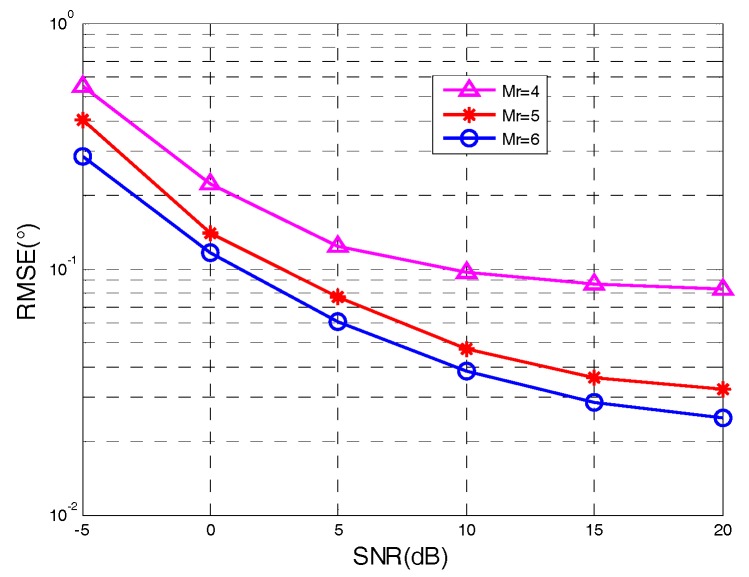
Angle tracking with different values of Mr.

Figure 6 and Figure 7 display the performance of angle tracking via our algorithm in the condition of P=2, L=100, F=200, SNR=10 dB, and variable numbers of M/N. It was clearly shown that the angle tracking performance of the proposed algorithm gradually improved with the increased number of transmit/receive antennas. Multiple transmit/receive antennas improved the angle tracking performance because of diversity gain. The correctness of the conclusions drawn from the theoretical error was validated.

## 6. Conclusions

In this paper, we proposed a moving multi-target angle tracking algorithm for bistatic MIMO radar. The proposed algorithm obtained the linear relationship between the covariance matrix difference and the angle difference through the three approximate processes. The proposed algorithm reduced the computational complexity and realized the automatic association of DOA and DOD. The proposed algorithm made full use of the elements of the covariance matrix by taking the average method, eliminating the noise, and improving the tracking performance. The research in this paper provides technical support for the practical application of the MIMO radar. In future work, we will analyze wideband signal processing to improve performance [25,26] and study signal processing in complex backgrounds to increase the robustness of the algorithm.
